# Identification of diagnostic biomarkers in Alzheimer’s disease by integrated bioinformatic analysis and machine learning strategies

**DOI:** 10.3389/fnagi.2023.1169620

**Published:** 2023-06-26

**Authors:** Boru Jin, Xiaoqin Cheng, Guoqiang Fei, Shaoming Sang, Chunjiu Zhong

**Affiliations:** ^1^Department of Neurology, Zhongshan Hospital, Fudan University, Shanghai, China; ^2^Collaborative Innovation Center for Brain Science, Fudan University, Shanghai, China; ^3^Shanghai Raising Pharmaceutical Technology Co., Ltd. Shanghai, China

**Keywords:** Alzheimer’s disease, biomarker, diagnosis, machine learning (ML), Bioinformatics

## Abstract

**Background:**

Alzheimer’s disease (AD) is the most prevalent form of dementia, and is becoming one of the most burdening and lethal diseases. More useful biomarkers for diagnosing AD and reflecting the disease progression are in need and of significance.

**Methods:**

The integrated bioinformatic analysis combined with machine-learning strategies was applied for exploring crucial functional pathways and identifying diagnostic biomarkers of AD. Four datasets (GSE5281, GSE131617, GSE48350, and GSE84422) with samples of AD frontal cortex are integrated as experimental datasets, and another two datasets (GSE33000 and GSE44772) with samples of AD frontal cortex were used to perform validation analyses. Functional Correlation enrichment analyses were conducted based on Gene ontology (GO), Kyoto Encyclopedia of Genes and Genomes (KEGG), and the Reactome database to reveal AD-associated biological functions and key pathways. Four models were employed to screen the potential diagnostic biomarkers, including one bioinformatic analysis of Weighted gene co-expression network analysis (WGCNA)and three machine-learning algorithms: Least absolute shrinkage and selection operator (LASSO), support vector machine-recursive feature elimination (SVM-RFE) and random forest (RF) analysis. The correlation analysis was performed to explore the correlation between the identified biomarkers with CDR scores and Braak staging.

**Results:**

The pathways of the immune response and oxidative stress were identified as playing a crucial role during AD. Thioredoxin interacting protein (TXNIP), early growth response 1 (EGR1), and insulin-like growth factor binding protein 5 (IGFBP5) were screened as diagnostic markers of AD. The diagnostic efficacy of TXNIP, EGR1, and IGFBP5 was validated with corresponding AUCs of 0.857, 0.888, and 0.856 in dataset GSE33000, 0.867, 0.909, and 0.841 in dataset GSE44770. And the AUCs of the combination of these three biomarkers as a diagnostic tool for AD were 0.954 and 0.938 in the two verification datasets.

**Conclusion:**

The pathways of immune response and oxidative stress can play a crucial role in the pathogenesis of AD. TXNIP, EGR1, and IGFBP5 are useful biomarkers for diagnosing AD and their mRNA level may reflect the development of the disease by correlation with the CDR scores and Breaking staging.

## Introduction

Alzheimer’s disease (AD) is the most prevalent form of dementia accounting for 60–80% of all cases (Alzheimer’s Association, 2022), and is becoming one of the main causes of death and posing a huge burden on patients and their families (Katzman, 2008; Georges et al., 2020). It is reported that about 50% of people aged 80 suffer from this disorder (Nalbantoglu et al., 2005) and the number of that would accumulate up to 115 million by 2050, which means there are 7.7 million increased cases every year and one more suffers every 4 s (Sosa-Ortiz et al., 2012). AD is manifested by memory loss, executive dysfunctions, and other cognitive deficits affecting patients’ ability to perform everyday activities (Querfurth and Laferla, 2010; Mckhann et al., 2011) and would eventually lead to the premature death of an individual occurring typically 3–9 years after diagnosis (Querfurth and Laferla, 2010). Due to the lack of effective treatments and the increasing average lifespan, AD has posed an enormous burden on worldwide economics and health (Alzheimer’s Association, 2016; Robinson et al., 2017).

The major neuropathological features of AD are intracellular neurofibrillary tangles (NFTs) formed by hyperphosphorylated tau protein, extracellular senile plaques composed of aggregated β-amyloid (Aβ) fibers (Tabaton et al., 1991; Smith, 1998), and progressive brain atrophy causing by loss of synapses and neurons (Butterfield and Halliwell, 2019; Alzheimer’s Association, 2021). Recently, attention has also been paid to other pathological markers including insulin resistance (de la Monte, 2017; Rad et al., 2018), oxidative stress (Perry et al., 2008; Jiang et al., 2016), neuroinflammation (Calsolaro and Edison, 2016), erythrocytic abnormality (Kosenko et al., 2020), mitochondrial dysfunction (Lustbader, 2004; Carvalho et al., 2019), and so forth. Several novel hypotheses were proposed such as the erythrocytic hypothesis (Kosenko et al., 2020), heart failure link to AD (Tublin et al., 2019), synaptic failure hypothesis (Tublin et al., 2019), and mitochondrial cascade hypothesis (Swerdlow et al., 2014). However, lacking a comprehensive understanding of the whole mechanism, none of these could precisely connect all the pathological events. There is an urgent need to detangle the mechanism of AD and identify useful biomarkers for diagnosis.

Bioinformatics analysis has evolved into an integrative field between computer science and biology, which allows the representation, storage, management, analysis, and investigation of numerous data types with diverse algorithms and computational tools (Mulder et al., 2017; Auslander et al., 2021). However, due to the quick development of next-generation sequencing and other emerging omics techniques, accumulated omics data at an astonishing speed and scope is urging for more effective approaches to conduct sophisticated analyses from various biomolecular levels, such as genomics, transcriptomics, proteomics, radiomics and metabolomics (Pevsner, 2015; Ayyildiz and Piazza, 2019). Fortunately, machine learning meets omics and exhibits extreme power in processing and modeling omics data with huge and diverse volumes (Li et al., 2022). Machine learning is a branch of artificial intelligence focusing on simulating human learning by exploring patterns in the data and applying self-improvement to continually enhance the performance of learning tasks (Auslander et al., 2021). Recently, the integrated bioinformatic analysis combined with machine-learning strategies was applied to the identification of potential pathways and diagnostic biomarkers of diseases, which has earned some praise (Journal of Nature Genetics, 2019; Auwul et al., 2021; Tran et al., 2021).

In our study, we integrated four frontal cortical datasets from the GEO database to discover novel pathways and identify diagnostic biomarkers of AD by bioinformatic analysis combined with machine learning strategies. The differential expressions and diagnostic efficacy of the identified biomarkers were verified in another two frontal cortical datasets of AD. The correlation analysis between the identified biomarkers and the CDR scores and Braaking staging. Finally, biomarkers associated with the key functional pathways in AD were identified and verified, which could also reflect the development of AD.

## Materials and methods

**FIGURE 1:**
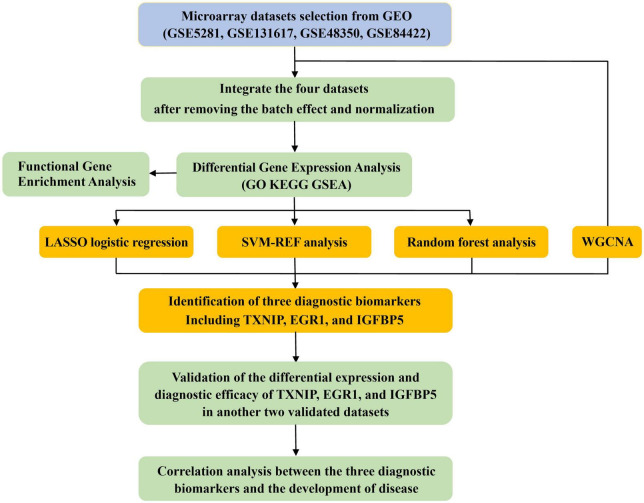
The workflow of the analysis process.

A diagram of the workflow of the bioinformatics analyses combined with machine learning strategies is shown in Figure 1.

### Data collection and data processing

We retrieved and downloaded six microarray expression profile datasets with the frontal cortex of AD patients from the National Center for Biotechnology Information (NCBI) Gene Expression Omnibus (GEO) database.^1^ The search was conducted with the following keywords: (“Alzheimer’s disease” and “Expression profiling by array”), and the species was restricted as “Homo sapiens.” Four datasets (GSE5281, GSE131617, GSE48350, and GSE84422) from the platform of Affymetrix are used as experimental datasets, and another two datasets (GSE33000 and GSE44772) from the platform of Rosetta/Merck were used as validation datasets.

Initially, referring to the annotation from the platforms, probes were annotated with gene symbols. Once there are multiple probes associated with the same gene, the average value of the expression would be calculated and applied. Then, the batch effect of the four experimental datasets was removed using the “Combat” function of the “SVA” package in R to fulfill normalization (Johnson et al., 2007). Finally, the validation of the differential expressions and diagnostic efficacy of identified biomarkers was performed on the validation datasets.

### Differential gene expression analysis

After normalization, four datasets were merged into an integrated dataset including 87 frontal cortial samples of AD and 126 controls. The differential gene expression analysis was conducted on this integrated dataset with the “limma” package in R. The | log2FC| (fold change) >2 and adjusted *p* < 0.05 were regarded as thresholds for the screening. Heatmaps and volcano plots were performed with “pheatmap” and “EnhancedVolcano” packages in R.

### Functional enrichment analysis

Focusing on all genes instead of only DEGs and demonstrating significantly enriched functional pathways more intuitively, gene set enrichment analysis (GSEA) was performed in R with clusterProfiler (Subramanian et al., 2005; Yu et al., 2012). Gene ontology (GO) enrichment analysis was conducted considering three hierarchical categories of biological process, molecular function, and cellular component with the “clusterProfiler” package in R (Yu et al., 2012). Pathway enrichment analysis was performed on Kyoto Encyclopedia of Genes and Genomes (KEGG) and Reactome database with “clusterProfiler” and “ReactomePA” packages in R (Yu et al., 2012; Yu and He, 2016; Minoru et al., 2017; Bijay et al., 2019).

### Screening the diagnostic biomarkers

Four models were applied to screen the potential diagnostic biomarkers, including one bioinformatic analysis and three machine-learning algorithms. Weighted gene co-expression network analysis (WGCNA) is a bioinformatic method describing the correlation between genes and sample traits, which has been widely used for identifying candidate biomarkers or therapeutic targets (Langfelder et al., 2009). The least absolute shrinkage and selection operator (LASSO) is a shrinkage and variable selection method for regression models, which was applied to identify the diagnostic genes associated with discrimination with the “glmnet” package in R (Ranstam and Cook, 2018). Support vector machine-recursive feature elimination (SVM-RFE) was conducted in R using the “e1071” package with fivefold cross-validation (Sanz et al., 2018). Random forest (RF) analysis was performed in R with the “randomForest” package (Rigatti, 2017). Finally, the Venn diagram was plotted to visualize the overlapping potential biomarkers among the four models as the candidate biomarkers (Bardou et al., 2014).

### Validation of the candidate biomarkers

The differential expression of the candidate biomarkers was verified in the validation datasets of GSE33000 and GSE44772. The diagnostic efficacy of the candidate biomarkers was evaluated by receiver operating characteristic (ROCs) analysis with the area under the curves (AUCs) (Seshan et al., 2013). The correlation analysis was performed to explore the correlation between the candidate biomarkers with CDR scores and Braak staging.

## Results

### Identification of differentially expressed genes

**FIGURE 2:**
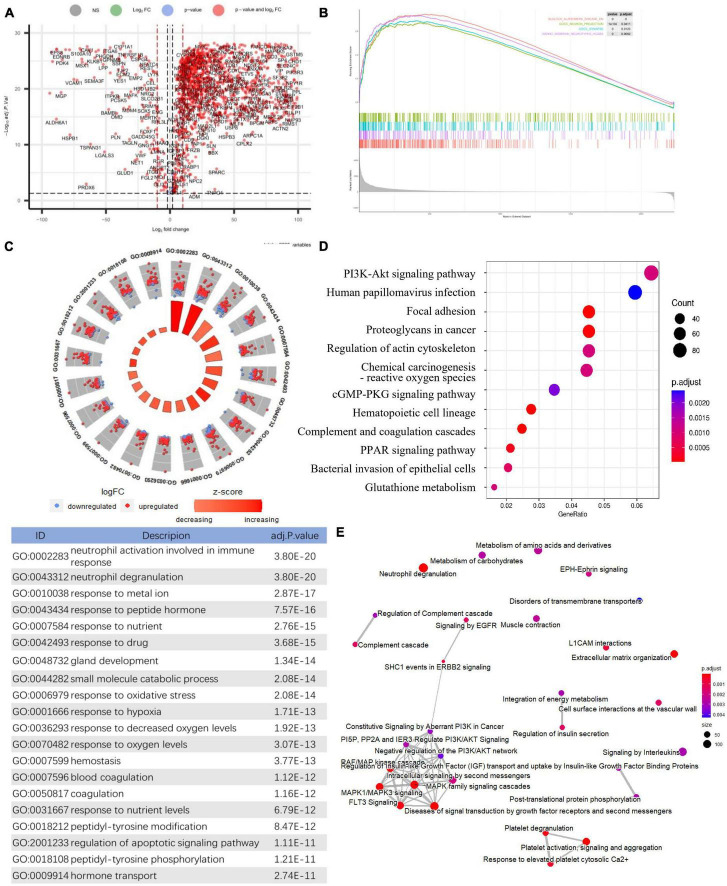
Identification of differentially expressed genes and Functional gene enrichment analyses. **(A)** Volcano plot; **(B)** GSEA profiles depicting the four significant GSEA sets in Alzheimer’s disease; **(C)** GO analyses results of DEGs. **(D)** KEGG pathway analysis of DEGs. **(E)** Reactome pathway analysis of DEGs.

After normalization, an integrated dataset with frontal cortical samples of AD was formed by four GEO datasets (GSE5281, GSE131617, GSE48350, and GSE84422), consisting of 87 AD patients and 126 control subjects. In the integrated dataset, the differential gene expression analysis identified 2235 DEGs, including 2029 downregulated genes and 206 upregulated genes in AD compared to the matched controls (Figure 2A and Supplementary Table 1). The visualized DEG expressions in the integrated dataset were shown in the heatmap (Supplementary Figure 1).

### Functional gene enrichment analyses

Gene set enrichment analysis in all detectable genes showed that genes in AD were mainly enriched in the following pathways: AD, neuron projection, synapses, multiple midbrain neurotypes, and so forth (Figure 2B). Also, GSEA_GO analysis revealed consistent results in pathway identification with GSEA analysis, in which dendrite and glutamatergic synapses were involved (Supplementary Table 1). GSEA_KEGG analysis showed that neurodegeneration and oxygen-related pathways were involved in AD, including the HIF-1 signaling and oxidative phosphorylation pathways (Supplementary Table 1). The results of the above GSEA analyses were further confirmed by GO, KEGG, and Reactome analysis on DEGs (Supplementary Table 1). The biological processes associated with immune response, oxidative stress, and apoptosis were significantly enriched by GO analysis (Figure 2C). PI3K-Akt signaling pathway was highlighted in both KEGG and Reactome analysis (Figures 2D, E).

### Screening the diagnostic biomarkers

**FIGURE 3:**
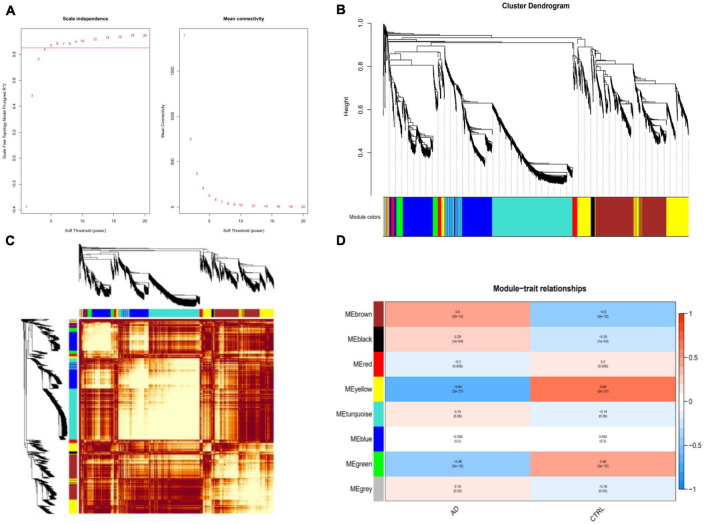
Weighted co-expression network analysis (WGCNA). **(A)** The scale-free fit index and the mean connectivity for various soft-thresholding powers of WGCNA. The left panel shows the scale-free fit index (*y*-axis) as a function of the soft-thresholding power (*x*-axis). The right panel displays the mean connectivity (degree, *y*-axis) as a function of the soft-thresholding power (*x*-axis). **(B)** Clustering dendrogram of differentially expressed genes related to Alzheimer’s disease, with dissimilarity based on the topological overlap, together with assigned merged module colors and the original module colors. **(C)** Heatmap depicts the Topological Overlap Matrix (TOM) of genes selected for WGCNA. Light color represents lower overlap and red represents higher overlap. **(D)** Relationships of consensus modules with diseases. Each specified color represents a specific gene module.

**FIGURE 4:**
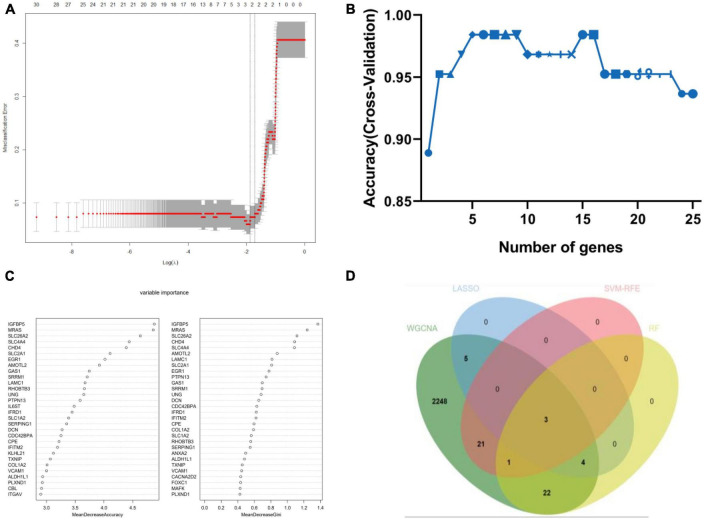
Machine-learning strategies for biomarker identification. **(A)** The cross-verification curve of least absolute shrinkage and selection operator (LASSO) logistic regression. **(B)** Support vector machine-recursive feature elimination (SVM-RFE) analysis. **(C)** Random forest (RF) analysis. **(D)** Venn diagram showed the intersection of diagnostic markers obtained by the four algorithms.

Weighted gene co-expression network analysis showed that eight remarkable co-expression gene modules identified were significantly correlated with AD (Figures 3A–D). LASSO logistic regression algorithm screened twelve potential diagnostic markers from DEGs (Figure 4A). SVM-RFE and RF analyses showed that there were 25 and 30 potential diagnostic markers associated with AD (Figures 4B, C). Among these potential biomarkers, there were three overlapping genes: thioredoxin interacting protein (TXNIP), early growth response 1 (EGR1), and insulin-like growth factor binding protein 5 (IGFBP5) (Figure 4D and Supplementary Table 1).

### Validation of the candidate biomarkers

**FIGURE 5:**
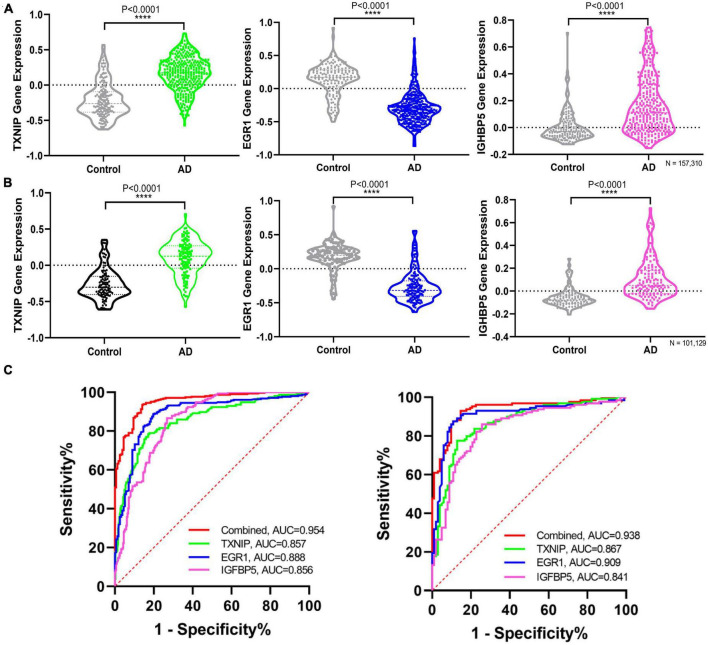
Validating the differential expression and diagnostic efficacy of the identified biomarkers. **(A)** Validation of the expression levels of the identified biomarkers in dataset GSE33000. **(B)** Validation of the expression levels of the identified biomarkers in dataset GSE44770. **(C)** Validation of the diagnostic efficacy of diagnostic biomarkers revealed by ROC analysis in the validation dataset GSE33000 and GSE44770 (TXNIP, EGR1, IGFBP5, and the combination of the three genes as a diagnostic tool).

**FIGURE 6:**
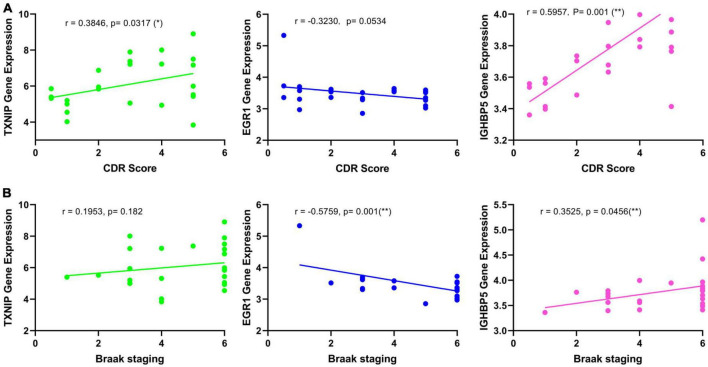
Correlation analysis of the identified biomarkers with the development of AD. **(A)** Correlation of the identified biomarkers with the CDR scores. **(B)** Correlation of the identified biomarkers with the Braak staging.

The expression changes of TXNIP, EGR1, and IGFBP5 were further validated in another two datasets GSE33000 and GSE44770. The results were consistent with the integrated dataset, in which TXNIP and IGFBP5 were significantly upregulated and EGR1 was downregulated (Figures 5A, B). The ROC analysis showed that the AUCs of TXNIP, EGR1, and IGFBP5 were 0.857, 0.888, and 0.856 in dataset GSE33000, 0.867, 0.909, and 0.841 in dataset GSE44770. The AUCs of the combination of these three biomarkers as a diagnostic tool for AD were 0.954 and 0.938 (Figure 5C). The correlation analysis indicated that TXNIP and IGFBP5 expressions were significantly correlated with CDR scores, and EGR1 and IGFBP5 expressions were significantly correlated with the Braak staging (Figure 6).

## Discussion

The frontal cortex has always been viewed as the “motor” lobe associated with two cognitive functions of memory and motor (Fuster, 1993; Boyle, 2004; Hashimoto et al., 2017) and is very vulnerable to suffering from impairment in AD. And studies suggested that the frontal cortex is quite sensitive to subclinical changes which may help predict cognitive impairments and early disturbance of daily activities (Stoeckel et al., 2013; Marshall et al., 2019). Hence, we select the frontal cortical samples of AD to identify the diagnostic biomarkers. To enlarge the scale of the sample size and simultaneously avoid the batch effect, we integrated four AD frontal cortical datasets of transcriptome from the same platform to conduct the analyses, and another two frontal cortical datasets to perform further validation. Considering the extreme power of machine learning in processing and modeling omics data, the integrated bioinformatic analysis was combined with three machine-learning strategies to fulfill the identification of diagnostic biomarkers.

The differential gene expression analysis revealed 2235 DEGs in AD compared to the matched controls, including 2029 downregulated genes and 206 upregulated genes (Figure 2A). Multiple functional enrichment analyses found that several essential pathways were significantly enriched in AD patients, including AD, neurodegeneration, synapse, immune response, oxidative stress, apoptotic signaling pathway, and so forth. It is widely known that the loss of synapse correlates the best with cognitive impairment and even precedes neuronal loss in AD, and there are many factors contributing to synaptic dysfunction in AD, especially the above-identified: immune response and oxidative stress (DeKosky and Scheff, 1990; Britschgi and Wyss-Coray, 2007; Hong et al., 2016). Mounting studies show that oxidative stress can impair synapses and contribute to AD through most pathological hypotheses including the amyloid cascade hypothesis, tau hypothesis, inflammatory hypothesis, and so forth (Ansari and Scheff, 2010; Zhao and Zhao, 2013; Bai et al., 2022). Accumulating evidence has also stressed that immune responses involving glial cells and the complement system are prominently activated in the AD brain, which can prune excess synapses inappropriately and mediate synapse loss eventually (Akiyama et al., 2000; Wyss-Coray, 2006; Britschgi and Wyss-Coray, 2007; Hong et al., 2016; Rajendran and Paolicelli, 2018). Generally, our results confirmed the pathological pathways of synapse and apoptosis in AD and further stressed the crucial role of the immune response and oxidative stress in the pathogenesis of the disease.

Moreover, one bioinformatic analysis of WGCNA and three machine-learning strategies of LASSO, SVM-RFE, and RF analyses commonly identified that TXNIP, EGR1, and IGFBP5 could serve as biomarkers of AD, combining them as a tool gave rise to high AUCs of 0.954 and 0.938 in the two verification datasets (Figure 5C). The correlation analysis further revealed that the expressions of TXNIP and IGFBP5 were significantly correlated with the CDR scores, and the expressions of EGR1 and IGFBP5 were significantly correlated with the Braak staging (Figure 6).

Previous studies have shown that TXNIP as an endogenous inhibitor of antioxidant thioredoxin was found to increase in AD patients and AD mouse models, and could be a key coordinator of different pathological processes (Tsubaki et al., 2020). TXNIP connects oxidative stress and inflammation by interaction with the nucleotide-binding domain, leucine-rich-containing family, and pyrin domain-containing-3 (NLRP3) inflammasome complex (Wang et al., 2019; Eraky and Ramadan, 2022; Sbai et al., 2022). Recent studies also suggested that blocking the interaction of NLRP3 provides a significant effect, and thus TXNIP could serve as a therapeutic target (Zhang et al., 2021). Therefore, TXNIP closely associated with the identified pathways of immune response and oxidative stress can be a useful biomarker of AD. EGR1 has also been reported in previous gene-wide association analyses using brain expression data (Koldamova et al., 2014; Mukherjee et al., 2017; Lim et al., 2018), which was associated with Aβ toxicity and was invalided in a C. elegans model (Mukherjee et al., 2017). Functionally, EGR1 helps maintain the brain’s cholinergic function during AD by regulating acetylcholinesterase (AChE) (Hu et al., 2019). EGR1 can bind to the BACE1 promoter and block the activation of the APP signaling to ultimately suppress the Aβ deposition and improve the cognitive function of AD (He et al., 2022). Studies also proposed EGR1 to be a key molecule affecting the activity of the nucleus basalis of Meynert by regulating synaptic activity and plasticity during AD (Zhu et al., 2016). Conclusively, EGR1 plays an important role in the development of AD and can serve as a useful biomarker. IGFBP5 is a pluripotent growth factor supporting neuronal survival and axon growth (Caroni and Grandes, 1990; Bach et al., 2005; Fernandez and Torres-Alemán, 2012; Rauskolb et al., 2017), which can coordinate the bioavailability and bioactivity of insulin-like growth factor 1 (IGF-1). IGFBP5 can modulate lipid metabolism and insulin sensitivity (Xiao et al., 2020), which are both associated with the cognitive impairment (Kao et al., 2020; Kellar and Craft, 2020). Studies have shown that IGFBP5 was associated with faster cognitive decline (Yu et al., 2018; Kim et al., 2019) and was found to increase in the brains (Rauskolb et al., 2022), cerebrospinal fluid (Salehi et al., 2008), and animal models of AD (Barucker et al., 2015).

Together, TXNIP, EGR1, and IGFBP5 served as potential biomarkers for AD diagnosis reflecting different pathogenetic pathways involved in the development of AD, which may be due to the complicated and multiple pathophysiological manifestations of AD. Besides the EGR1-associated Aβ deposition which has gained the most concern, our results suggested that the TXNIP-associated pathways of the immune response, oxidative stress, and especially their interaction should be paid more attention. More importantly, the identification of IGFBP5 highlights the role of insulin metabolism in the pathogenesis and development of AD. Evidence from epidemiological, clinical, and neuropathology has shown that patients with diabetes are at higher risk of developing AD due to impaired brain insulin signaling (Barbiellini Amidei et al., 2021; De Felice et al., 2022). Studies have repurposed anti-diabetes agents as novel therapeutics for AD, while how impaired insulin signaling and brain insulin resistance occurs remains unclear, urging further exploration.

There are some limitations of our study. Firstly, although we tried to select the same region of frontal cortex in AD brains from the same platform and have performed validation in another two verification datasets, the results still need more experimental confirmation for the data is from publicly available microarray datasets. Secondly, given the limited scale of sample size and type, the diagnostic efficacy of biomarkers should be further explored clinically, and even in samples of blood and cerebrospinal fluid. Thirdly, we fail to identify the early detecting biomarkers of AD due to the lack of datasets on patients with mild cognitive impairment (MCI), though early detection is the now most urgent need under the huge burden of increasing incidence and heavy cost. And we would like to perform the analysis of identifying the potential diagnostic biomarkers of MCI, once there were available datasets.

## Conclusion

The integrated bioinformatic analysis combined with machine learning strategies can effectively help identify the functional pathways and diagnostic biomarkers in disease. Based on these methods, we stressed the crucial roles of immune response and oxidative stress in the pathogenesis of AD and identified three genes associated with the above two pathways as useful biomarkers, including TXNIP, EGR1, and IGFBP5. Furthermore, the expressions of TXNIP, EGR1, and IGFBP5 may reflect the development of AD by correlation with the CDR scores and Braak staging.

## Data availability statement

The data presented in the study are deposited in the Gene Expression Omnibus (http://www.ncbi.nlm.nih.gov/geo) repository, accession numbers GSE5281, GSE131617, GSE48350, GSE84422, GSE33000, and GSE44772.

## Author contributions

SS and CZ designed the study and accessed the funding. BJ and XC downloaded the data and performed the bioinformatics analysis. SS, GF, and BJ wrote the manuscript. All authors approved the submitted manuscript.

## Footnotes

1
http://www.ncbi.nlm.nih.gov/geo
