# Supplementary figures and images for: Identification of diagnostic biomarkers in Alzheimer’s disease by integrated bioinformatic analysis and machine learning strategies
Source: Front Aging Neurosci. 2023 Jun 26;15:1169620. doi: 10.3389/fnagi.2023.1169620 (PMC10331604; doi:10.3389/fnagi.2023.1169620)

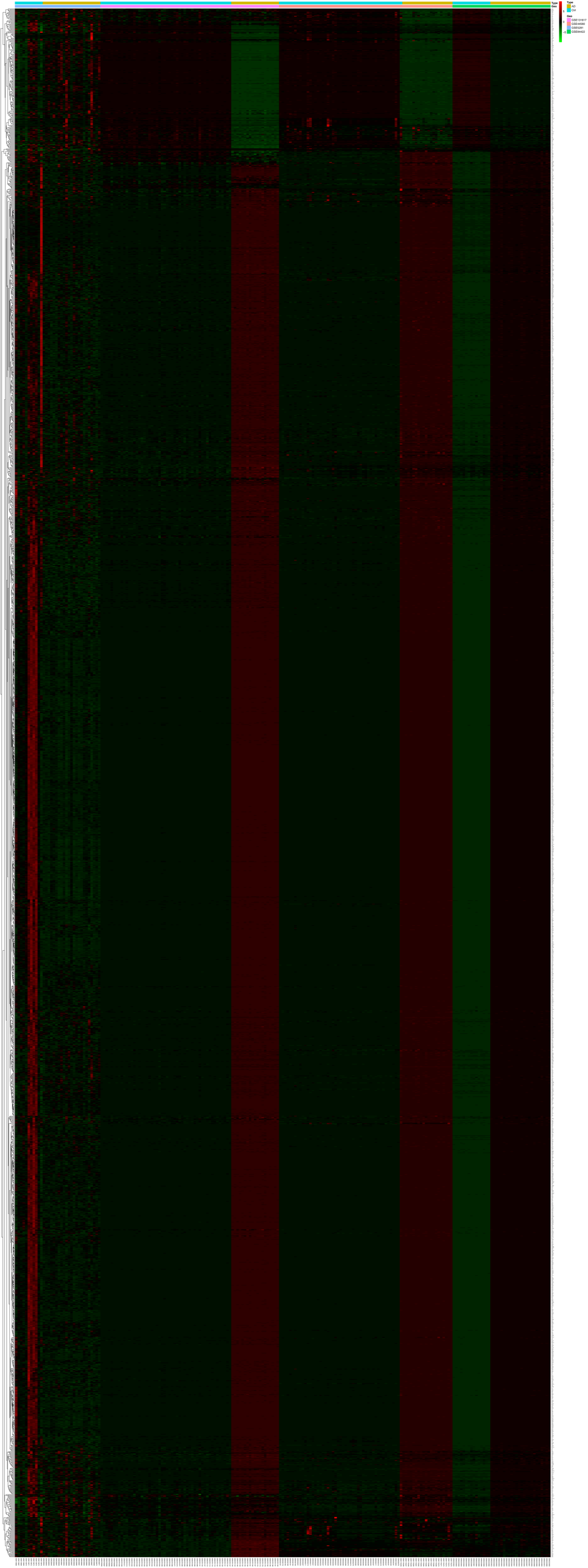

Supplement: Supplementary file 1 [file Data_Sheet_1.PDF]
